# Prognostic value of neutrophil to lymphocyte ratio in gastric cancer patients receiving immune checkpoint inhibitors: a systematic review and meta-analysis

**DOI:** 10.3389/fonc.2023.1070019

**Published:** 2023-04-18

**Authors:** Siheng Zhang, Chao Qiu, Hanzhi Yu, Yan Xu, Xiaoming Xu

**Affiliations:** ^1^ Zhejiang Provincial Center for Disease Control and Prevention, Hangzhou, Zhejiang, China; ^2^ School of Public Health, Jinan University, Guangzhou, Guangdong, China; ^3^ School of Basic Medical Sciences, Lanzhou University, Lanzhou, China; ^4^ Department of Pharmacology, School of Basic Medical Science, Tianjin Medical University, Tianjin, China; ^5^ Department of Gastroenterology, Jining First People's Hospital, Jining, China

**Keywords:** gastric cancer, meta-analysis, neutrophil to lymphocyte ratio (NLR), biomarker, immune checkpoint inhibitors (ICIs)

## Abstract

**Background:**

The neutrophil to lymphocyte ratio (NLR) is a cost-effective and easily identifiable inflammatory biomarker that has been shown to be closely associated with tumor prognosis and predict survival in patients with multiple malignancies. However, the predictive value of NLR in patients with gastric cancer (GC) treated with immune checkpoint inhibitors (ICIs) has not been fully explored. Therefore, we conducted a meta-analysis to explore the potential of NLR as a predictor of survival in this population.

**Methods:**

We systematically searched the PubMed, Cochrane Library, and EMBASE databases from inception to the present for observational researches on NLR and its relationship with progression or survival in GC patients receiving ICIs. To assess the prognostic significance of NLR on overall survival (OS) or progression-free survival (PFS), we used fixed or random-effect models to derive and combine hazard ratios (HRs) with 95% confidence intervals (CIs). We also examined the relationship between NLR and treatment efficacy by calculating relative risks (RRs) with 95% CIs for objective response rate (ORR) and disease control rate (DCR) in patients with GC receiving ICIs.

**Results:**

Nine studies of 806 patients were eligible. OS and PFS data were obtained from 9 and 5 studies, respectively. In nine studies, NLR was associated with poor survival, the pooled HR was 1.98 (95% CI 1.67- 2.35, p < 0.001), indicating a significant association between high NLR and worse OS. We conducted subgroup analyses based on study characteristics to confirm the robustness of our findings. A relationship between NLR and PFS were reported in five studies with a HR of 1.49 (95% CI 0.99- 2.23, p = 0.056), which was not significantly associated. Pooling four studies that examined the correlation between NLR and ORR/DCR in GC patients, we observed a significant correlation between NLR and ORR (RR = 0.51, p = 0.003), but no significant correlation between NLR and DCR (RR = 0.48, p = 0.111).

**Conclusion:**

In summary, this meta-analysis indicates that increased NLR is significantly linked to worse OS in patients with GC receiving ICIs. In addition, lowering NLR can improve ORR. Thus, NLR can serve as a predictor for prognosis and treatment response in GC patients treated with ICIs. Nevertheless, further high-quality prospective studies are required to verify our findings in the future.

## 1 Introduction

According to projections from the International Agency for Research on Cancer (IARC), by 2020 there would be 1,089,103 new cases of gastric cancer (GC) globally, accounting for 5.6% of all diagnosed cancer cases, with 768,793 deaths attributable to the disease (1). GC remains the fourth most frequent type of cancer, with a high mortality rate (2). Conventional therapies have limited clinical efficacy, and the median overall survival rate for advanced GC is only approximately 8 months (3). Over the past decade, immune checkpoint inhibitors (ICIs) with monoclonal antibodies that suppress programmed cell death protein 1 (PD-1), PD-L1, and cytotoxic T lymphocyte antigen 4 (CTLA-4) has emerged as a promising therapeutic option for various cancers (4). After surgery, chemotherapy, radiation, and targeted therapy, immunotherapy has become an effective treatment technique and one of the breakthroughs in cancer treatment (5). ICIs can effectively interrupt the interaction of immune checkpoints, thereby disrupting tumor cells by activating the host’s immune system. Compared with traditional therapies, immune therapy has demonstrated potent efficacy and tolerable toxicity (6).

Chronic inflammation is linked to various steps of tumorigenesis, including cell transformation, invasion, proliferation, and angiogenesis (7). The systemic inflammatory response plays a significant role in the origin, progression, and metastasis of cancer and has a bearing on the clinical outcomes of cancer patients (8, 9). Tumor cells and associated inflammatory cells release large amounts of cytokines, chemokines, and other inflammatory factors at different stages of tumor development, invasion, and metastasis, promoting tumor cell growth (10). Proven tumor-induced systemic inflammatory responses have been found to be effective prognostic biomarkers in many cancers. For example, a low lymphocyte-to-monocyte ratio (LMR) before therapy is related to advanced clinicopathological characteristics and poor prognosis in individuals with pancreatic cancer (11). In numerous malignancies, including hepatocellular carcinoma and colorectal cancer, the neutrophil-to-lymphocyte ratio (NLR) in peripheral blood has been shown to have a prognostic relationship (12, 13). NLR is a simple and conveniently obtained biomarker that can measure the inflammatory status of the immune system.

ICIs are an important component of current GC treatment, particularly for advanced stage patients. Multiple studies have shown that both ICIs monotherapy and combined strategies with chemotherapy or other therapies significantly improve the survival of advanced GC patients (14–17). In patients who are Epstein-Barr virus (EBV) positive or microsatellite instability (MSI) positive, ICI has shown better response rates (18, 19). In Japan, nivolumab is now licensed for the treatment of patients with advanced stomach cancer who are resistant to standard chemotherapy. However, more inexpensive and convenient markers are needed to predict the efficacy and response to immunotherapy. Currently, there is no meta-analysis examining the predictive significance of NLR and its changes in GC patients treated with ICIs.

Therefore, we included retrospective or prospective cohort studies comparing the difference in prognosis and treatment response between high and low NLR for patients with advanced or locally advanced GC treated with ICIs to investigate the prognostic value of NLR for this group of patients.

## 2 Methods and materials

### 2.1 Search strategy

This meta-analysis was conducted following the Preferred Reporting Items for Systematic Reviews and Meta-Analyses (PRISMA) statement (20). Two independent researchers conducted a search of PubMed, Embase, and Cochrane Library to identify relevant papers on the prognosis of NLR in GC patients treated with immune checkpoint inhibitors, from inception to July 15, 2022. The following search terms were used to investigate the predictive significance of NLR and ICIs in patients with GC: (“neutrophil-lymphocyte ratio” OR “neutrophil-to-lymphocyte ratio” OR NLR) AND (“gastric cancer” OR “gastric adenocarcinoma”) AND (“PD-L1 inhibitor” OR “immune checkpoint inhibitor” OR “programmed death ligand-1 inhibitor” OR “immunotherapy”). The search terms were slightly modified for different databases. In addition, references of selected articles were screened to avoid missing any relevant studies.

### 2.2 Inclusion and exclusion criteria

Articles that met the following criteria were included (1): studies on patients with histopathologically confirmed advanced or locally advanced gastric cancer, (2) studies reporting long-term survival data, including overall survival (OS) or progression-free survival (PFS), objective response rate (ORR), or disease control rate (DCR), or provided data sufficient to calculate these outcomes, (3) studies published in English, and (4) studies reporting hazard ratios (HRs) or relative risks (RRs) with 95% confidence intervals (CI), either directly or obtained from the original research. The following exclusion criteria were applied: (1) studies reporting on the predictive significance of inflammatory markers without specific information on NLR, (2) studies without sufficient data, (3) conference abstracts, letters, editorials, expert opinions, reviews, and case reports.

### 2.3 Data extraction

Two researchers independently extracted the subsequent data from each article, and inconsistencies were resolved via discussion or consultation with a third researcher: first author, publication year, study country, study design, total number of cases and NLR value, subject age (mean or median), HR for OS and PFS with corresponding 95% CI, and ORR or DCR data.

### 2.4 Study quality assessment

The Newcastle-Ottawa Scale (NOS) was used to assess study quality. Two researchers independently scored eight questions each, on a scale of 0-9. Studies scoring more than 6 points were considered of high quality (21).

### 2.5 Statistical methods

Statistical analysis was performed using Review Manager 5.3 and STATA 14.0 software. RR were used to assess the relationship between NLR and ORR or DCR in patients with gastric cancer. HR and their associated 95% CI were used to evaluate possible associations of NLR with OS and PFS. Heterogeneity between studies was assessed by the Cochran’s Q-test and I^2^, and appropriate effect models were selected based on them. Random effect models were used when I^2^ > 50% or p-value < 0.10 (for the Q-test) indicated significant heterogeneity. Otherwise, fixed effect models were used. We evaluated publication bias by observing the symmetry of the funnel plot, as well as by Begg regression and Egger’s linear regression methods, and p-values > 0.05 were deemed as indicative of no publication bias. We also conducted sensitivity analyses to determine the influence of each study on OS and PFS, and eventually, we calculated pooled statistics.

## 3 Results

### 3.1 Literature search results

**Figure 1:**
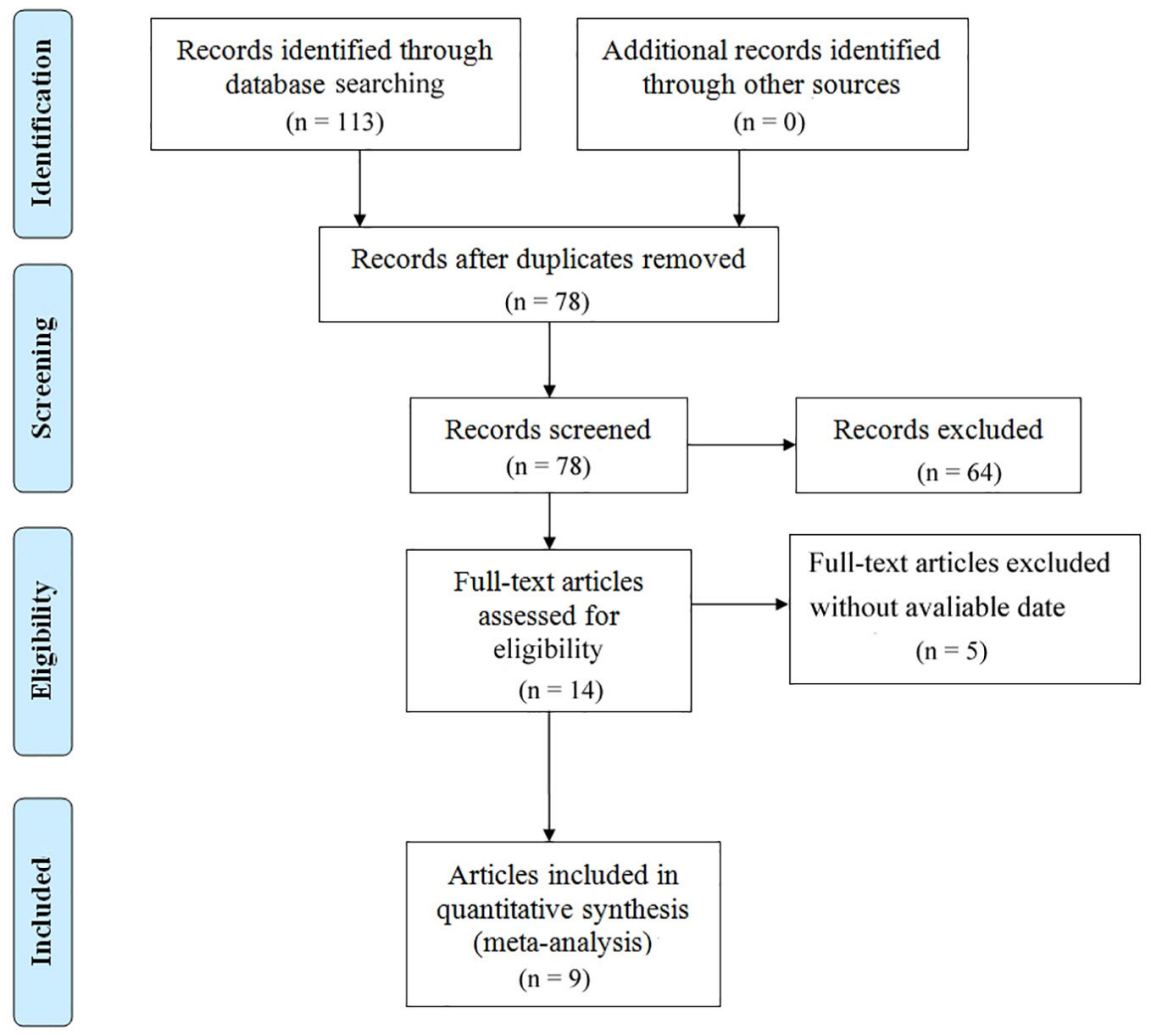
PRISMA flow chart for selection and inclusion of eligible studies.

The process of literature selection was illustrated in **Figure 1**. At the outset, 113 studies were identified through database searches. Upon screening the titles and abstracts, 64 studies were excluded as they failed to meet the inclusion criteria, including duplicate reports, conference abstracts, reviews, and case reports. Five articles were also excluded as full text could not be obtained. Finally, nine observational cohort studies, including eight retrospective and one prospective study, were included in the meta-analysis, totaling 806 patients (22–30). The main features of the included studies were summarized in **Table 1**. The studies were published since 2014, with most conducted in Japan. The sample sizes ranged from 26 to 185, with seven studies using only Nivolumab and the remaining two studies using multiple ICIs that included Nivolumab. All included articles had NOS scores as shown in **Table 2**. Overall, the quality of the data was sufficient to explore the prognostic significance of NLR in patients with GC receiving ICIs therapy.

**Table 1:** Baseline characteristics of all included studies.

Study, Year	Country	Duration	Study design	Sample size	Age	Gender (M/F)	Follow-up (months)	ICIs	Cut-off	Survival outcome	Analysis	NOS
Ogata 2018	Japan	2017	Retrospective	26	median: 64	19/7	median: 5.7	Nivolumab	5	OS, PFS	U, U	7
Namikawa 2020	Japan	2017-2019	Retrospective	29	median: 71	19/10	median: 32	Nivolumab	2.5	OS, PFS	U, U	7
Ota 2020	Japan	2014-2018	Retrospective	98	median: 66	68/30	median: 4.9	Nivolumab	3	OS, PFS	M, U	8
Suzuki 2020	Japan	2017-2019	Retrospective	72	NR	57/15	median: 4.8	Nivolumab	5	OS	M	8
Yamada 2020	Japan	2014-2019	Retrospective	89	NR	42/47	median: 5.83	Nivolumab	2.5	OS, PFS	M, M	8
Gou 2021	China	2016-2020	Retrospective	137	median: 59	98/39	NR	Nivolumab Pembrolizumab Toripalimab Sintilimab	3.23	OS, PFS	M, M	7
Kim 2021	Korea	2016-2019	Retrospective	185	median: 59	120/65	median: 4.8	Nivolumab Pembrolizumab	3	OS	U	7
Tanaka 2021	Japan	2017-2019	Prospective	70	median: 69	46/24	12	Nivolumab	5	OS	M	8
Sakai 2022	Japan	2017-2020	Retrospective	100	median: 71	78/22	median: 5	Nivolumab	2.54	OS	M	8

M, male; F, female; NR, not report; ICIs, immune checkpoint inhibitors; OS, overall survival; PFS, progression-free survival; U, univariate; M, multivariate; NOS, Newcastle-Ottawa Scale.

**Table 2:** Quality assessment using the Newcastle-Ottawa Scale (NOS).

Studies	Selection				Comparability	Outcome			Scores
	A	B	C	D	E	F	G	H	
Ogata 2018	★	★	★	★	★	★	★	–	7
Namikawa 2020	★	★	★	★	★	★	★	–	7
Ota 2020	★	★	★	★	★★	★	★	–	8
Suzuki 2020	★	★	★	★	★★	★	★	–	8
Yamada 2020	★	★	★	★	★★	★	★	–	8
Gou 2021	★	★	★	★	★★	★	–	–	7
Kim 2021	★	★	★	★	★	★	★	–	7
Tanaka 2021	★	★	★	★	★★	★	★	–	8
Sakai 2022	★	★	★	★	★★	★	★	–	8

A study may receive a maximum of one star for each numbered item in the Selection and Outcome categories. A maximum of two stars may be given for Comparability, as directed by the NOS.

### 3.2 Relationship of primary outcome measure (OS) and secondary outcome measure (PFS) to NLR

**Figure 2:**
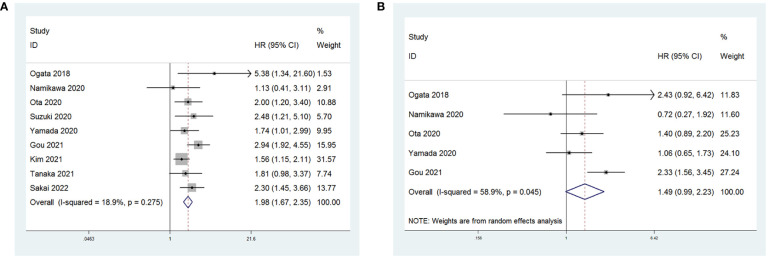
Forest plot for the association between NLR and **(A)** overall survival (OS) and **(B)** progression-free survival (PFS).

All nine studies reported on the relationship between NLR levels and OS in GC patients treated with ICIs. After conducting a heterogeneity test, the results showed no heterogeneity (P = 0.275 > 0.1, I^2^ = 18.9% < 50%), indicating that a fixed-effects model was appropriate for the meta-analysis. The pooled HR was 1.98 (95% CI: 1.67 to 2.35, P < 0.001), suggesting that higher NLR values were associated with worse OS in GC patients **(** **Figure 2A** **)**.

Five studies reported on the relationship between NLR levels and PFS in GC patients receiving ICIs. A random-effects model was used due to considerable heterogeneity among the included studies (P = 0.045 < 0.1, I^2^ = 58.9%). The combined HR was 1.49 (95% CI: 0.99 to 2.23, P = 0.056). However, the association between an increase in NLR and PFS in patients receiving ICIs was not statistically significant (**Figure 2B**).

### 3.3 Assessment of publication bias

**Figure 3:**
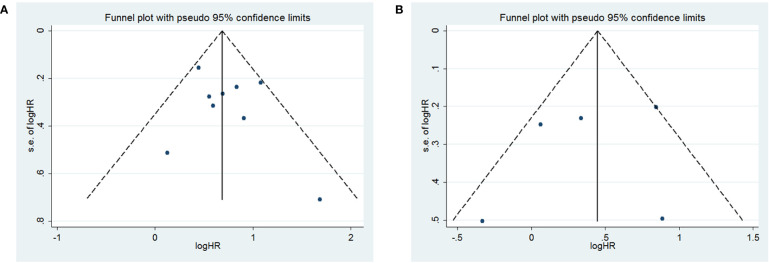
Funnel plot for **(A)** overall survival (OS) and **(B)** progression-free survival (PFS).

**Figure 4:**
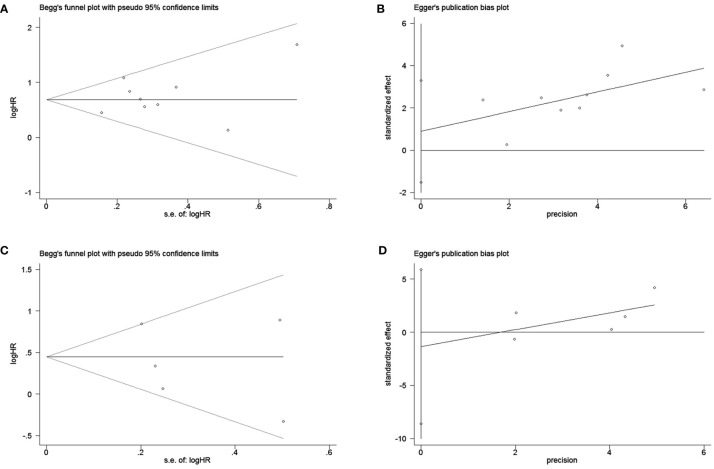
Begg and egger test for overall survival (OS) **(A, B)**; Begg and egger test for progression-free survival (PFS) **(C, D)**.

To assess publication bias, HRs and their associated 95% CIs for OS and PFS were aggregated and evaluated using a funnel plot and the Begg and Egger tests. The funnel plots for both OS and PFS showed good symmetry **(** **Figure 3A** for OS and **Figure 3B** for PFS**)**. The Begg test (p = 1.0 for OS, p = 0.462 for PFS) and Egger test (p = 0.412 for OS, p = 0.597 for PFS) indicated that there was no significant publication bias for OS (**Figures 4A, B**) and PFS (**Figures 4C, D**).

### 3.4 Assessment of sensitivity analysis

**Figure 5:**
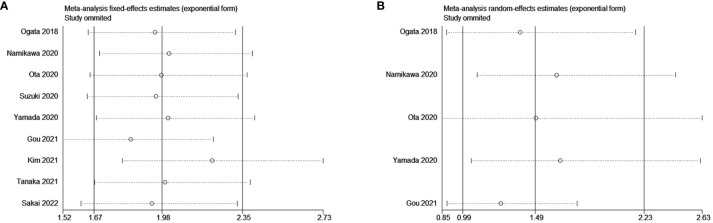
Sensitivity analysis for the association between NLR and **(A)** overall survival (OS) and **(B)** progression-free survival (PFS).

Sensitivity analyses showed no significant effect of any study on the observed effect size for the association between NLR and OS and PFS. Furthermore, no significant change occurred by removing any of the articles in this study, which indicates that the random-effects model used above was stable. (**Figure 5A** for OS and **Figure 5B** for PFS).

### 3.5 Subgroup analysis

To ascertain the origin of OS heterogeneity, we conducted a subgroup analysis. Our findings indicate that high-NLR indicated worse OS among patients, regardless of publication country (China, Japan, or Korea), sample size (≥ 100 or < 100), cut-off value (> 3 or ≤3), or analytical model (multivariate or univariate) (**Table 3**).

**Table 3:** Results of subgroup analysis for impact of NLR on overall survival.

Subgroup	NO. of studies	Hazard Ratio (95% CI)	P	Heterogeneity		Model
				I2 (%)	Ph	
Country						
China	1	2.94 (1.91-4.52)	<0.001	–	–	–
Japan	7	2.03 (1.60-2.58)	<0.001	0	0.649	Fixed
Korea	1	1.56 (1.15-2.12)	0.004	–	–	–
Sample size						
≥100	3	2.14 (1.44-3.18)	<0.001	66.7	0.05	Random
<100	6	1.94 (1.48-2.56)	<0.001	0	0.574	Fixed
Cut-off						
>3	4	2.60 (1.91-3.54)	<0.001	0	0.439	Fixed
≤3	5	1.76 (1.43-2.16)	<0.001	0	0.582	Fixed
Analysis						
Univariate	3	1.60 (1.20-2.13)	0.001	41.2	0.183	Fixed
Multivariate	6	2.24 (1.80-2.77)	<0.001	0	0.683	Fixed

95% CI, 95% confidence interval; Ph, p value of Q for heterogeneity test.

### 3.6 Association between NLR and ORR/DCR

**Figure 6:**
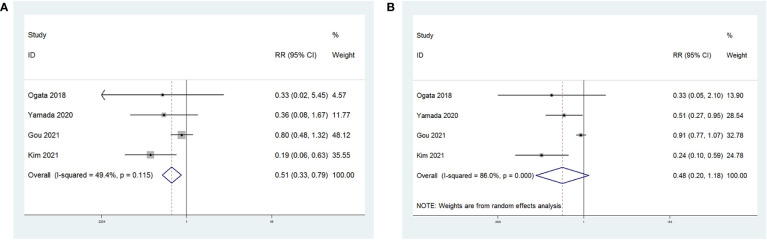
Forest plot for the association between NLR and **(A)** objective regression rate (ORR) and **(B)** disease control rate (DCR).

In four of these studies, shown in **Figure 6**, the relationship between NLR and therapy effectiveness (ORR or DCR) in patients with GC receiving ICIs was investigated. NLR and ORR had a significant relationship (RR = 0.51; p = 0.003). However, NLR and DCR had no significant relationship (RR = 0.48; p = 0.111).

## 4 Discussion

Gastric cancer is the fourth most common cancer worldwide and the second leading cause of cancer-related deaths (31, 32). In addition to genetic factors, the incidence of GC can also be attributed to various pathogenic infections, resulting in high morbidity and mortality (33, 34). Despite the progress made in multimodal therapy for GC, recurrence of the disease is still common (35). Furthermore, owing to the absence of early diagnostic markers, GC is frequently diagnosed in its advanced stages, significantly diminishing the likelihood of survival, dependable biomarkers are critically necessary to facilitate early detection and survival forecasting.

Thus, many studies have investigated related inflammatory factors and tumor prognosis to use biological indicators to predict survival outcomes after a certain treatment and provide timely intervention to improve patient survival rates (36, 37). Among the inflammatory factors, NLR has been widely studied by scholars because of its low cost and easy availability. Some previous studies have researched whether NLR can predict the prognosis of GC. Han et al. demonstrated that pre-operative NLR is an independent prognostic factor in patients with GC and were associated with worse survival (38). Hirahara et al. found that the outcome of treatment and prognosis in patients with advanced gastric cancer can be predicted by the combination of NLR and platelet-to-lymphocyte ratio (PLR) (39). Wang et al. demonstrated that pretreatment NLR could be a prognostic factor for survival in locally advanced gastric cancer receiving adjuvant chemotherapy after D2 resection (40).

While several studies have evaluated the predictive value of NLR in patients, to our knowledge, no meta-analysis has yet comprehensively examined whether NLR predicts survival outcomes in GC patients receiving ICIs. To address this gap in knowledge, we conducted a meta-analysis of data from nine relevant trials involving 806 patients from three countries to determine whether survival outcomes could be predicted by NLR values in gastric cancer patients treated with ICIs. Our analysis found that higher NLR values were associated with a lower survival rate and a significant correlation existed between high NLR and poor OS, with the combined HR of NLR and OS being 1.98. In addition, reducing NLR increased ORR, while high NLR played a negative role in ORR in patients treated with ICIs. However, the relationship between NLR and PFS was not statistically significant (p = 0.056).

It is important to note that our study has several limitations. First, all studies included in this analysis were conducted on gastric cancer patients in Asian countries, with seven of the studies coming from Japan. Although subgroup analyses did not reveal significant differences between the studies in the three countries, the regionalization of the studies suggests caution should be applied in extrapolating the results to Western countries due to potential differences in biology. Second, the majority of the included studies were retrospective, which would have resulted in a reduced level of evidence. Finally, some studies with relatively small sample sizes may have introduced selection bias.

## 5 Conclusion

In conclusion, our meta-analysis suggests that a higher NLR is significantly correlated with worse OS and adverse ORR in GC patients treated with ICIs. NLR may serve as a promising biomarker for predicting prognosis and treatment response. However, more large-scale, multicenter, high-quality prospective trials are required to validate our findings.

## Data availability statement

The original contributions presented in the study are included in the article/supplementary material. Further inquiries can be directed to the corresponding author.

## Author contributions

XX, CQ and YX contributed to the research concept and design. CQ and HY retrieved and filtered articles, CQ and SZ extracted data. CQ and SZ analyzed the data. SZ, CQ, XX, and YX explained the data. CQ and SZ drafted manuscript. SZ, XX and YX contributed to critical revision of the manuscript. All authors contributed to the article and approved the submitted version.
